# A Cross-Sectional Study to Explore Intimate Partner Violence and Barriers to Empowerment of Women in Armenia

**DOI:** 10.1155/2019/6939684

**Published:** 2019-07-16

**Authors:** Russell Kabir, Hafiz T. A. Khan

**Affiliations:** ^1^School of Allied Health, Anglia Ruskin University, UK; ^2^The Graduate School, University of West London, UK

## Abstract

**Background:**

Intimate partner violence is a major problem worldwide and it is one of the most social issues in Armenia. Empowerment is one of the important factors that helps women to break the cycle of violence by their husband/partner. The aim of this research is to explore the impact of intimate partner violence on empowerment of Armenian women of reproductive age group.

**Methods:**

This cross-sectional study used data Armenia Demography and Health Survey Data 2015-16. A total 6116 women were selected from 8749 households at both urban and rural places of Armenia for interview using multistage cluster sampling technique. Data analysis was performed using SPSS version 24.

**Results:**

The respondents aged between 35 and 49 years are more likely to face violence compared to other age group (p≤0.001). The respondents who have no decision-making power, about 89% of them, are experiencing intimate partner violence, whereas only 11% are facing intimate partner violence among those who have decision-making power (p≤0.001). The logistic regression analysis reveals that age of the respondents, number of children in the households, wealth index, and empowerment status are significantly associated with intimate partner violence.

**Conclusion:**

Intimate partner violence has significant impact on the empowerment of women in Armenia. This study revealed that women with no empowerment are more likely to experience intimate partner violence compared to those women who are empowered in Armenian society.

## 1. Introduction

Violence against women is considered as most inexorable human rights violation in the world [1]. It is common source of physical, psychological, and emotional morbidity [2]. According to the recent statistics, worldwide about 30% women aged 15 and over have experienced physical and sexual violence by their partners and only 7% women have faced sexual violence by nonpartners in their lifetime [3, 4]. Violence against women can happen within marriage, long-term relationships, or short-term relationships and be committed ex-partners when the relationship ends [5]. Around the world, women suffer from intimate partner violence, marital rape, rape by other men known to them and by strangers, incest, foeticide, sexual harassment, trafficking for prostitution and forced labour, dowry related violence, honour killings, other forms of femicide, acid attacks, and female genital mutilation, and these are all considered as gender-based violence as men are committing them against women [6].

Violence against women is one of the rampant human right violations [7]. Violence against women is the most common but least punished crime in the world and it has been estimated that globally women aged 15 to 44 are more likely to be maimed or die as a result of male violence than cancer, malaria, traffic accidents, and war combined [8]. Violence against women is violation of basic human rights that must be eliminated through political will and by legal and civil action in all sectors of society [9].

Intimate partner violence is currently accepted term to describe “women abuse”, “violence against women”, “domestic violence”, or “women battering” [2]. In this research the term “intimate partner violence”, “domestic violence”, and “violence against women” interchangeably used. Intimate Partner violence refers to actual or threatened psychological, physical or sexual harm by a current or former partner or spouse [10]. Intimate partner violence as rape, physical assault, and stalking perpetrated by current former dates, spouses, and cohabiting partners [11].

Women's empowerment has become an important issue for development efforts worldwide [12]. Empowerment is a personal, multidimensional phenomenon and difficult to measure directly [13]. Empowerment is defined as to the expansion in peoples' ability to make strategic life choices in a context where this ability was previously denied to them [14]. There are several indicators of empowerment at the level of individual women and her household as formulated: (i) participation at crucial decision-making process; (ii) sharing of domestic activities with men; (iii) women taking control of her reproductive function and decides on family size; (iv) women being able to decide where the income she has earned will be channelled to; (v) feeling and expression of pride and value in her work; (vi) self-confidence and self-esteem; and (vii) ability to prevent violence [15].

Women's empowerment consists of decision-making power, control over financial issues, and sexual empowerment [16]. Women's degrees of empowerment are defined by gender and gender relations in the society and gender does not always mean biological sex; it is the different roles, rights, and obligations that are attached by society to individuals born with male or female sex characteristics [17]. Global development efforts depend on gender equality and recommended that promoting gender equality helps the economy to grow. Intimate partner violence (IPV) is considered as a serious indicator of disempowerment of women [16, 18]. Studies have indicated that women who have experienced intimate partner violence are associated with women's empowerment variable [19]. Women's limited employment opportunities and lack of access to inheritance and income prevents them leaving violent relationship [20].

Women's limited decision-making power and with constrained economic resources can inhibit them to accessing health care services and this may finally lead to serious health complications [21]. IPV rate is declining as women's economic role is expanding and they are getting stronger sense of their rights [22]. Economic empowerment is not the only sole factor, together with higher education and changed cultural models will help women from IPV [23]. Empowerment does not necessarily lower the odds that a woman experienced spousal violence and the relationship between women's empowerment to decreased or increased spousal violence is context specific [24].

Armenia a nation of Euroasia with a population of 3 million, gained its independence after the dissolution of the USSR in 1991 [25]. Like any newly independent nation, Armenia suffered incredible economic calamity and living condition worsened in the years immediately after the independence [26] and women lost their jobs, they became resilient and adaptable to the changing circumstances [27]. Women are at more risk of violence during and after times of conflict and security and it has been found that more than one-third of married women have experienced have faced physical or sexual type of violence in 7 out 13 conflict and insecure countries [28].

Violence against women remains one of the most serious social issues in Armenia and violence at household level to be widespread [29]. Armenian security forces recorded 784 violence report and each year the organisation receives more than 2000 calls about domestic violence cases but there is still high rate of under reporting of violence against women so the actual number of women who face violence at home is significantly very high [30]. Furthermore, the domestic violence cases were not properly prosecuted which had detrimental impact on women [31]. The women in Armenia are very family oriented and they serve a major role in conserving cultural values in the family and caring for children and the household [32]. Culturally, the women in Armenia are very respectful to men whether it is husband, father, father in law or brother and poor economic status particularly unemployment increases family tension and violence against women. Very few of them will talk about their family problems with an outsider [33]. It has been found that violence against women is strongly associated with the status of women in the society and use of violence is considered as exercising power on women [23]. Abolishing all forms of violence against women was adopted as a target for the Sustainable Development goals (SDG) 5 on gender equality and empowerment of women [34]. Despite the emerging body of literature on this subject area there is limited evidence on how violence against women affect their empowerment in Armenia. On this backdrop, this research is trying to explore the impact of intimate partner violence on empowerment of women in Armenia. This research aims to explore the relationship of violence and empowerment of women by incorporating other aspects of women's empowerment and the results are expected to help the policy-makers in Armenia.

## 2. Methodology

This descriptive cross-sectional study used data from a nationally representative Armenia Demographic and Health Survey 2015-16.

### 2.1. Sampling and Sample Size

The sampling frame used in this survey is a complete list of enumeration areas (EA) covering the whole country and a total number of 11,571 EAs were selected using census database. Each EA in the frame is also subdivided into two types of residence –urban and rural. In rural areas, an EA is a natural village, a segment of a large village, or a group of small villages; in urban areas, an EA is a street or a city block. Of the total EAs, of the total of 11,571 EAs, 6,613 are in urban areas and 4,958 are in rural areas. Overall, each EA has an average of 69 households, with EAs in urban areas averaging 79 households and those in rural areas averaging 56 households [35].

A representative probability sample of 8,749 households was selected. The sample was selected in two stages: (i) for the first stage, 313(192 urban areas and 121 in rural areas) clusters were selected from a list of EAs and (ii) in the second stage, complete listing of households was performed from each selected cluster and then households were selected systematically for participation.

A total of 6116 women were selected for interview [35]. The following inclusion criteria are used to select the participants:

1. Women age between 15 and 49
2. Permanent residence of the households
3. Women who were present in the household on the night before the survey

### 2.2. Data Analysis and Variables

Statistical data analysis was performed using SPSS version 24. There are different types of inferential statistical tests but in this research chi-square and binary logistic regression analysis are performed. To estimate the association between dependent and independent variable bivariate logistic regression analysis is performed. The first dependent variable used in this research experience of intimate partner violence is categorical with category scale “Yes” and “No”. “Yes” stands for “experienced IPV” and “No” stands for “did not experience IPV” and the following logistic model is used:

(1) $$\begin{array}{c} \gamma =\log\left(\;\frac{\pi }{1-\pi }\;\right)=\beta_{0}+\beta_{1}x_{1}+\beta_{2}x_{2}+\ldots \beta_{m}x_{m} \end{array}$$

Here, *π*= stands for probability to experience IPV, 1-*π*= stands for probability not to experience IPV, *β*_0_ is the intercept term, and *β*_1_ s are logistic regression coefficients (i=1,2….,n).

The IPV variable was constructed by combining physical violence, sexual violence, and emotional violence. The physical violence was computed by respondent answering “yes” to any of a string questions about her partner/husband ever did the following: (i) ever been pushed, shook or had something thrown; (ii) ever been slapped; (iii) ever been punched; (iv) ever been dragged; (v) ever been threatened with knife/gun or other objects. The sexual violence variable was determined by ever been forced into unwanted sex by the respondent's husband/partner and the emotional violence was determined by respondent answering “yes” and “no” to ever experience emotional violence.

The empowerment variable was measured using the following questions: (i) person who usually decides how to spend respondent's earnings; (ii) person who usually decides on respondent's healthcare; (iii) person who usually decides on large household purchases; and (iv) person who usually decides on visits to family or relatives. Empowerment of women was recorded as “0” or “Yes” when the respondents replied, “respondent alone” and it was recorded as “1” or “No” when the respondents replied “husband/partner”.

There are two types of independent variables used in this research:

- Woman's characteristics
  - Place of residence and the categories are urban and rural.
  - Highest educational level was no education, basic education, and secondary and higher education.
  - Age of the respondents was categorised into 15 to 24 years, 25 to 34 years, and 35 to 49 years.
  - Number of children in the household was categorised into 1 child, 2-3 children, and more than 4 children.
  - Household members in the family are 1, 2 to 3, and more than 4.
  - Wealth index was categorised into poorest, poorer, middle, richer, and richest.
  - Employment status is categorised into two yes means who were employed during the time of this survey and no means who were not employed.
  - It owns a mobile phone: yes and no.
  - It has a bank account: yes and no.
  - Empowerment status has two categories: women who have decision-making power and women who have no decision-making power. The empowerment variable was computed by adding variables such as (a) person who usually decides how to spend respondent's earnings; (b) person who usually decides on respondent's healthcare; (c) person who usually decides on large household prices; (d) person who usually decides on visits to family or relatives; and (e) person who usually decides what to do with money husband earns. Women's participation in household decision-making reflects the interaction of various factors related to empowerment and therefore, these are strong measures of empowerment [24].
- Husband's characteristics
  - Husband's age was grouped to make categories such as 20 to 29 years, 30 to 39 years, 40 to 49 years, 50 to 59 years, and 60 years and above.
  - Husband's drinking alcohol is categorised into yes and no.
  - Husband/partner educational status is categorised into no education, basic education, and secondary and higher education.

### 2.3. Ethical Issues

The proposed research uses publicly available data and so no ethical approval is required from any Institutional Review Board.

### 2.4. Findings

Table 1 presents the background information about the respondents. About 58% of the participants are from urban areas for Armenia. About 42% respondents completed secondary education, 7 % have completed basic education, and 51% of them completed higher education. Approximately 27% respondents are between 15 to 24 years of age and 39% of them are between 35 to 49 years of age. The mean age of the respondents is 31.50 years. Almost 69% respondents have one child, 30% respondents have 2-3 children, and only 1% have more than 4 children. About 79% respondents revealed that they have more than four family members in their households and 20% of them have 2-3 family members. Only 33% respondents are employed during the time of the interview. A vast majority (97%) of the respondents use mobile phone and 29% of them use their mobile phone for business purposes. Only 8% respondents shared that their covered by the health insurance and 20% of them have a bank account. About 22% respondents are from poorer background, 18% respondents are categorised as poorest, and only 16% of them are from richest family. Only 10% respondents husband/partner completed primary education, 50% of them completed secondary, and 40% respondents husband/partner completed secondary special education. A vast majority of the (62%) respondents husband/partner drinks alcohol. The mean age of respondent's husband/partner is 39 years and approximately 36% respondent's husband/partner age is between 30 and 39 years and 32% respondent's husband/partner age is between 40 and 49 years.

Pearson chi-square analysis has been performed in Table 2 to assess the relationship between the characteristics of the respondents and intimate partner violence. The results show that women from urban area are more to suffer from intimate partner violence compared to women from rural but the chi-square test show that the results are not statistically significant. The women with higher and secondary education are more likely be abused by their husband/partner compared to women than women with no and basic education (p-value ≤0.001). The respondents' age between 35 and 49 years is more likely to face violence compared to the respondents from other age group (p- value ≤0.001). About 59% respondents with 1 child are more likely to experience IPV compared to respondents with 2 to 3 children. The women who are not employed about 65% of them are experiencing IPV compared to the respondents who are employed, and this is statistically significant. There are no significant differences observed among the different categories of wealth index with intimate partner violence. A vast majority 78% woman who have no bank account shared that they face more violence than those who have bank account with a p-value ≤ 0.001. The respondents who have no decision-making power, about 89% of them experiencing intimate partner violence compared to those women who decision-making power, only 11% of them are facing intimate partner violence (p-value ≤0.001). It also presents that respondents with husband/partner age between 30 and 39 are more likely to commit violence (p-value≤0.001). Comparing to the respondents whose partner/husband does not drink alcohol, this finding is statistically significant with a p-value ≤0.001. Also, education of husband/partner is an important factor and this study shows that husband/partner with secondary and secondary special education are more likely to abuse their wives/partners than husband/partner with no and primary education (p-value≤0.001).

In Table 3, logistic regression analysis highlighted the effects of women's background characteristics on intimate partner violence. Age group, children in the household, wealth index, and empowerment status had significant effects on intimate partner violence. Women in the age group 25-34 years had 0.39 times lower odds of experiencing inmate partner violence than those women who belonged to 15-24 years of age. Similarly, women who belonged to 25-34 years of age had 0.19 times lower odds of experiencing inmate partner violence than those who belonged to 15-24 years of age. Women who had 2-3 children in the household were 0.40 times less likely to experience inmate partner violence than those women who had one child in the household. Additionally, women who had more than 4 children in the household were 0.15 times less likely to experience inmate partner violence than those women who had one child in the household. Women who were socioeconomically rich had less chance to experience inmate partner violence than poor women. Women who did not have decision-making power had less chance to experience inmate partner violence than those women who had decision-making power. Husband's age and alcoholic husband/partner had significant effects on inmate partner violence. The probability of experiencing inmate partner violence among women was significantly higher for those whose husbands/partners were aged 60 and over than those whose husbands/partners belonged to age group 20-29 years. Women with alcoholic husbands/partners were 2.40 times more likely to experience inmate partner violence than those whose husbands/partners were not alcoholic.

## 3. Discussion 

Violence against women is a serious social, political, and public health issue in Armenia [36]. Evaluating woman's experience with violence is a difficult task, some women may tolerate more violence than others but due to fear they are reluctant reporting the violence. Research on intimate partner violence has been done before but the recent study focused on the Armenian women using a nationally representative sample. There is no empirical research conducted before on empowerment status of Armenian women and its relationship with IPV and the purpose of this study to fill out this gap in the literature. The research findings show that the mean age of respondents in this study is 31 years, about 38% of the respondents aged between 35 and 49 years, and only 42% of them completed secondary education. This is consistent with a recent research finding from Botswana where the mean age of the respondents was 32 years and majority had completed secondary education [37] and is like another research in Tanzania [38]. Older women within the 16-49 age range are less likely than younger women aged 16-49 to report being violently victimised by a partner [39]. About 30% women shared they have 2 to 3 children but in a Spanish study of 1402 randomly selected woman shared that 33.3% of them had two children [40]. Consistent with this study finding, a qualitative study with women victims of domestic violence in Armenia shows that majority of the respondents completed only 10 years of school education, had one or two children, were mainly from urban areas and were divorced [36]. This study also presents that only 32% women are employed, and this is contrast to the finding in the Nepal study where 65% respondents were unemployed [41].

The chi-square analysis results also indicated that intimate partner violence was more prevalent among women with higher education (52.3%) but different results are found in Southeast Nigeria, South Karnataka, Romania, European Union, Spain, Bangladesh, Pakistan, and Tanzania [42–50] where women with primary education were the most abused and women's education exhibit strongest association with IPV in a national survey in India [51]. Women's educational achievement can reduce the risk of intimate partner violence for women [52, 53]. Education of women is also an important factor and this study presents that educational level of women is associated with empowerment status of women; therefore women basic and higher education are more empowered but respondents from higher educational attainment are more likely to take their own decisions [13].

The logistic regression analysis explored the relationship between intimate partner violence with background characteristics of women and it shows that age of the respondents, number of children in the households, wealth index, and empowerment status are significantly associated with intimate partner violence. This study did not exhibit any relationship between place of residence, educational level, employment status, and household member with intimate partner violence. According to wealth index category, the women from richer socioeconomic background are less likely to suffer from poorer women but another research found that wealth index does not have any impact on domestic violence among the women of Pakistan but consistent with the findings from the research done in Tanzania [1, 54]. The advanced analysis also revealed that women with more than 4 children in the household are less likely to experience intimate partner violence.

This study revealed that women with no decision-making power are less likely to experience the violence comparing to the women who have decision-making power. This is opposite to the finding in Jordan where the authors found that the women who can take decision independently in the household matters and income related issues are less likely to suffer from intimate partner violence [55] and same reported in a study carried out in Pakistan [1] and the lifetime experience of spousal violence was very high among the women with low empowerment level in Nepal [24]. Further investigation is required for this to understand why the findings are different in the country from other places. This study disclosed that women in the age group 15 to 24 years are more likely to face violence by their husband/partner and similar result was found in Tanzania [54] but different result observed in Burkina Faso [56] and their analysis found that older women ag between 45-49 are more likely to participate in decision-making process. The difference observed in Armenia due to changing the cultural system and younger women are more independent and liberal in this 21st century whereas the older women are still scared of breaking the norms and speak against their partner/husband.

There are several strengths and limitations of this research. This research only focused on the experiences of the women, so it does not give a clear understanding why the violence takes place from the husband/partner's point of view. This research has used secondary data that has been collected to answer a different research question and did not match with the research question of this study. Another limitation of this research is the cross-section design used which does not established the causal relationship between empowerment of women and intimate partner violence in Armenia, so the conclusion is based on the relationship between dependent and independent measures used. Despite some limitations, this study poses some strengths. The study sample used in this research obtained from a nationally representative sample population, this is the first study to use nationally representative data and it is likely that findings can be generalised in Armenia and this is the first research to show empowerment of women and intimate partner violence using nationally representative data. It would have been superlative to gather the qualitative information from the participants thus making the findings of the research more suitable and meaningful.

## 4. Conclusion and Recommendations

Women empowerment is one of the important agendas in this 21st century for sustainable development of a country. There is not enough literature of violence against women and their empowerment status in Armenia and this research is going to serve as an important source of information for future researchers and policy-makers with the given field. Intimate partner violence has significant impact on the empowerment of women in Armenia. The conclusion which can be drawn from the current research is that there is room for improvement in the sociopolitical system of Armenian society and more reasonable approaches to tackle the intimate partner violence issue in Armenia could be created by upgrading the social infrastructure. The base for violence against women lies in the severely inscribed conception of patriarchy so change of a cultural mindset is very much necessary to stop it. The sociocultural model needed to be redefined and revaluated and legal system should be stricter so that the abuser could not escape easily, and a stronger legislation which is place will help to protect the women from getting abused in their homes. The government should create more social and legal help centres for the victims of violence at local and regional level and there should be provision for free legal support for the victims.

Policies should be created to reduce gender discrimination and women should be given equal opportunities to make equal contribution and participation in the social, political economic spheres of the country. Equal opportunity to women will not only benefit the both genders it will contribute massively for the upliftment of the society. A practical approach should be taken to challenge the violence against women at all levels of Armenian society and more public campaigns needed to increase the awareness about this social problem among the community. Education/outreach programs are beneficial for the people in Armenia specially the women. It is very important to educate the women of disadvantaged and lower socioeconomic status. Education will boost confidence level among women and will help them to be more independent and self-sufficient; also they will be more aware about the laws and legislation related to violence against women. Mass media can play a vital role here; by promoting and educating the women it can help to prevent and reduce the number of violence cases in the country. To move forward as a nation, significant changes required in the sociocultural structure of the country and it will only be achieved by creating general awareness among the masses about role of women and their empowerment and gender balancing in the society and by providing a positive and suitable for the women. More human rights programmes should be developed and implemented at local, regional, and community level to reach the deprived and marginalised community. More research is needed to be done to get a clearer picture of the conditions which make the women in Armenia more susceptible to violence and further research work needed to explore the perspectives of male counterpart who abuses their partners. Government and nongovernment organisations should take positive steps for reducing domestic violence and should organise different kind of programmes to educate and motivate women in Armenia to enhance their employment opportunities.

## Figures and Tables

**Table 1:** Background Characteristics of the respondents and characteristics of their husbands (n=6116).

Categories	Frequency	Percentage
*Place of Residence*		
Urban	3545	58
Rural	2571	42
*Highest educational level*		
No education	5	0.1
Basic	406	6.6
Secondary	2580	42.2
Higher	3125	51.1
*Age group*		
15-24	1665	27.2
25-34	2081	34
35-49	2370	38.8
Mean ±SD	31.50±9.5	
*Children in the household*		
1 child	4199	68.7
2-3 Children	1831	29.9
More than 4 children	86	1.4
*Household members*		
1	60	1
2-3	1239	20.3
More than 4	4817	78.8
*Employment Status*		
No	4108	67.2
Yes	2006	32.8
*Owns a Mobile phone*		
No	199	3.3
Yes	5917	96.7
*Use of Mobile phone for financial transaction*		
No	4215	71.3
Yes	1699	28.7
*Cover by Health Insurance*		
No	5637	92.2
Yes	478	7.8
*Has a bank account*		
No	4910	80.4
Yes	1199	19.6
*Wealth index*		
Poorest	1137	18.6
Poorer	1358	22.2
Middle	1324	21.6
Richer	1293	21.1
Richest	1004	16.4
*Husband educational level*		
No education	7	0.2
Primary	384	9.6
Secondary	1993	49.9
Secondary special	1608	40.3
*Age*		
20-29	612	15.3
30-39	1447	36.2
40-49	1283	32.1
50-59	636	15.9
60+	20	0.5
Mean ±SD	39.36±8.8	
*Drinks alcohol*		
No	1334	37.7
Yes	2204	62.3

**Table 2:** Crosstabulation of characteristics of respondents with intimate partner violence.

Background Characteristics	IPV		*p*-value
	Yes (%)	No (%)	

*Place of Residence*			
Urban	58.9	57	
Rural	41.1	43	≥0.12
*Highest educational level*			
No education	0.1	0.1	
Basic	4.7	8.6	
Secondary	42.9	41.4	
Higher	52.3	49.9	≤0.001
*Age group*			
15-24	10.5	43.6	
25-34	43.2	25	
35-49	46.3	31.4	≤0.001
*Children in the household*			
1 child	58.7	78.4	
2-3 Children	39.8	20.2	
More than 4 children	1.4	1.4	≤0.001
*Household members*			
1	0.6	1.3	
2-3	20	20.5	
4+	79.4	78.1	≤0.01
*Employment Status*			
No	65.1	69.2	
Yes	34.9	30.8	≤0.001
*Owns a Mobile phone*			
No	2.5	4	
Yes	97.5	96	≤0.001
*Cover by Health Insurance*			
No	90.6	93.7	
Yes	9.4	6.3	≤0.001
*Has a bank account*			
No	78.7	82	
Yes	21.3	18	≤0.001
*Wealth index*			
Poorest	17.4	19.8	
Poorer	21.4	23	
Middle	22.2	21.1	
Richer	22	20.3	
Richest	17	15.8	≤0.02
*Empowerment Status*			
Have decision making power	11	67	
Have no decision-making power	89	33	≤0.001

Husband/partner characteristics	IPV		*p*-value
	Yes	No	

*Age *			
20-29	15.2	15.7	
30-39	41.1	23.8	
40-49	29.4	38.8	
50-59	13.9	20.9	
60+	0.4	0.8	≤0.001
*Husband/partner drinks alcohol*			
No	40.8	19.3	
Yes	59.2	80.7	≤0.001
*Husband/partner education*			
No education	0.2	0.1	
Primary	9.4	10.0	
Secondary	48.2	54.2	
Secondary Special	42.1	35.7	≤0.001

**Table 3:** Odds ratio analysis between intimate partner violence and background characteristics of women and husband's characteristics.

Background Characteristics	ODDs Ratio (95% CI)	*p*-value

*Place of Residence*		
Urban	1	
Rural	0.98(0.77-1.27)	≥0.91
*Highest educational level*		
No education	1	
Basic	2.82(0.47-17.06)	>0.25
Secondary	1.48(0.27-8.84)	>0.67
Higher	1.45(0.24-8.73)	>0.68
*Age group*		
15-24	1	
25-34	0.39(0.30-0.49)	≤0.001
35-49	0.19(01.5-0.26)	≤0.001
*Children in the household*		
1 child	1	
2-3 Children	0.40(0.32-0.49)	≤0.001
More than 4 children	0.15(0.04-0.51)	≤0.001
*Household members*		
1	1	
2-3	1.04(0.56-1.90)	≥0.91
4+	1.10(0.60-2.02)	≥0.75
*Employment Status*		
No	1	
Yes	1.04(0.84-1.28)	>0.74
*Owns a Mobile phone*		
No	1	
Yes	1.25(0.72-2.15)	≥0.42
*Has a bank account*		
No	1	
Yes	1.12(0.86-1.45)	≥0.40
*Wealth index*		
Poorest	1	
Poorer	0.79(0.60-1.04)	≥0.09
Middle	0.71(0.52-0.97)	≤0.03
Richer	0.56(0.39-0.79)	≤0.001
Richest	0.55(0.39-0.79)	≤0.001
*Empowerment Status*		
Have decision-making power	1	
Have no decision-making power	0.047(0.039-0.057)	≤0.001

Husband characteristics	Odds Ratio (95% CI)	*p*-value

*Husband's age*		
20-29	1	
30-39	1.32(0.89-1.95)	≥0.16
40-49	1.92(1.31-2.85)	≤0.001
50-59	1.68(1.08-2.62)	≤0.02
60+	4.26(1.24-14.65)	≤0.02
*Husband/partner drinks alcohol*		
No	1	
Yes	2.40(1.86-3.13)	≤0.001

## Data Availability

These data were derived from the following resources available in the public domain, https://www.dhsprogram.com/.

## Abbreviations

EA: Enumeration area
IPV: Intimate partner violence
SDG: Sustainable development goals
SPSS: Statistical package for social science.

## References

[1] Ferdous N., Kabir R., Khan H. T. A., Khanchowdhury M. (2017). Exploring the relationship of domestic violence on health seeking behavior and empowerment of women in Pakistan. *Epidemiology, Biostatistics and Public Health*.

[2] Cronholm P. F., Fogarty C. T., Ambuel B., Suzanne L. H. (2011). Intimate partner violence. *American Family Physician*.

[3] Devries K. M., Mak J. Y., Garcia-Moreno C. (2013). The global prevalence of intimate partner violence against women. *Science*.

[4] Abrahams N., Devries K., Watts C. (2014). Worldwide prevalence of non-partner sexual violence: a systematic review. *The Lancet*.

[5] Tuiketei T., Rokoduru A. (2010). *Violence Against Women - A Public Health Perspective: Project Report Fiji 2010*.

[6] Johnson H., Ollus N., Nevala S. (2007). *Violence against Women: An International Perspective*.

[7] Oxford International (2012). *Ending Violence against Women: An Oxfam Guide*.

[8] UN (2007). *International Women’s Day Fact Sheet on Violence Against Women. International Women’s Day 2007. Take action to end impunity for violence against women and girls*.

[9] Modiba L., Baliki O., Mmalasa R., Reineke P., Nsiki C. (2011). Pilot survey of domestic abuse amongst pregnant women attending an antenatal clinic in a public hospital in Gauteng Province in South Africa. *Midwifery*.

[10] Weil A. (2016). *Intimate Partner Violence: Epidemiology and Health Consequences*.

[11] Tjaden P. G., Thoennes N. Extent, nature, and consequences of intimate partner violence.

[12] Upadhyay U. D., Gipson J. D., Withers M. (2014). Women's empowerment and fertility: A review of the literature. *Social Science & Medicine*.

[13] Kabir R., Rahman S., Monte-Serrat D. M., Arafat S. Y. (2017). Exploring the decision-making power of bangladeshi women of reproductive age: Results from a national survey. *South East Asia Journal of Medical Sciences*.

[14] Kabeer N. (2001). Reflections on the measurement of women's empowerment. *Discussing Women’s Empowerment-Theory and Practice. Sida Studies No. 3*.

[15] Medel-Anonuevo C. (1995). Women, education and empowerment: pathways towards autonomy. *UIE Studies*.

[16] Kwagala B., Nankinga O., Wandera S. O., Ndugga P., Kabagenyi A. (2016). Empowerment, intimate partner violence and skilled birth attendance among women in rural Uganda. *Reproductive Health*.

[17] Kishor S., Subaiya L. (2008). *Understanding Womens Empowerment: A Comparative Analysis of Demographic and Health Surveys (DHS) Data*.

[18] World Bank (2012). *World Development Report 2012: Gender Equality and Development*.

[19] Sethuraman K., Lansdown R., Sullivan K. (2006). Women's empowerment and domestic violence: the role of sociocultural determinants in maternal and child undernutrition in tribal and rural communities in South India. *Food and Nutrition Bulletin*.

[20] Murshid N. S. (2017). An assessment of the association between asset ownership and intimate partner violence in Pakistan. *Public Health*.

[21] Ahmed S., Creanga A. A., Gillespie D. G., Tsui A. O. (2010). Economic status, education and empowerment: implications for maternal health service utilization in developing countries. *PLoS ONE*.

[22] Schuler S. R., Lenzi R., Nazneen S., Bates L. M. (2013). Perceived decline in intimate partner violence against women in Bangladesh: qualitative evidence. *Studies in Family Planning*.

[23] Dalal K. (2011). Does economic empowerment protect women from intimate partner violence?. *Journal of Injury and Violence Research*.

[24] Tuladhar S., Khanal KR., Lila KC., Ghimire PK., Onta K. (2013). *Womens Empowerment and Spousal Violence in Relation to Health Outcomes in Nepal: Further Analysis of The 2011 Nepal Demographic and Health Survey*.

[25] Sevoyan A., Agadjanian V. (2010). Male migration, women left behind, and sexually transmitted diseases in Armenia. *International Migration Review*.

[26] Thompson M. E., Harutyunyan T. L. (2006). Contraceptive practices in Armenia: panel evaluation of an information-education-communication campaign. *Social Science & Medicine*.

[27] Armenia A. D. B. (2015). *Country Gender Assessment*.

[28] Head S. K., Zweimueller S., Marchena C., Hoel E. (2014). *Womens Lives and Challenges: Equality and Empowerment since 2000*.

[29] CEDAW Task Force (2016). *State parties on the implementation of the convention on the elimination of all forms of discrimination against women*.

[30] Coalition to stop violence against women Femicide in Armenia: A Silent Epidemic. http://coalitionagainstviolence.org/en/femicide-en/.

[31] Minnesota Advocates for Human Rights Domestic Violence in Armenia. http://www.mnadvocates.org/.

[32] Amoros Z., Callister L., Sarkisyan K. (2010). Giving birth: the voices of Armenian women. *International Nursing Review*.

[33] Mkrtchyan I. Violence against women in Armenia.

[34] Jewkes R., Fulu E., Tabassam Naved R. (2017). Women’s and men’s reports of past-year prevalence of intimate partner violence and rape and women’s risk factors for intimate partner violence: A multicountry cross-sectional study in Asia and the Pacific. *PLoS Medicine*.

[35] National Statistical Service [Armenia] Ministry of Health [Armenia], and ICF. Armenia Demographic and Health Survey 2015-16. Rockville, Maryland, USA: National Statistical Service, Ministry of Health, and ICF.

[36] Sardaryan Y., Thompson M. E., Kagan S., Truzyan N. Domestic violence from the perspective of women supported by shelters and crisis centers in armenia: a qualitative study.

[37] Dougherty D., Winter S. C., Haig A. J., Ramaphane P., Barchi F. (2018). Intimate partner violence and women's health-seeking behaviors in Northwestern Botswana. *Journal of Health Care for the Poor and Underserved*.

[38] Msuya S. E., Adinan J., Mosha N. Intimate partner violence and empowerment among women in Tanzania: Prevalence and effect on utilization of reproductive and maternal health services.

[39] Mattingly M. J. (2005). *After He Hits Her... Consequences of Intimate Partner Violence [Doctoral dissertation]*.

[40] Ruiz-Perez I., Plazaola-Castano J., del Rio-Lozano M. (2007). Physical health consequences of intimate partner violence in Spanish women. *European Journal of Public Health*.

[41] Shrestha M., Shrestha S., Shrestha B. (2016). Domestic violence among antenatal attendees in a Kathmandu hospital and its associated factors: a cross-sectional study. *BMC Pregnancy and Childbirth*.

[42] Anolue F. C., Uzoma O. I. (2017). Intimate partner violence: prevalence, contributing factors and spectrum among married couples in Southeast Nigeria. *International Journal of Reproduction, Contraception, Obstetrics and Gynecology*.

[43] Tomy C., Mani M. R., Deepa S., Christy S. A., Johnson A. R. (2018). Intimate partner violence experienced by pregnant women availing antenatal care at a rural hospital in South Karnataka. *International Journal of Community Medicine and Public Health*.

[44] Rada C. (2014). Violence against women by male partners and against children within the family: prevalence, associated factors, and intergenerational transmission in Romania, a cross-sectional study. *BMC Public Health*.

[45] Reichel D. (2017). Determinants of intimate partner violence in Europe: the role of socioeconomic status, inequality, and partner behavior. *Journal of Interpersonal Violence*.

[46] Vives-Cases C., Torrubiano-Domínguez J., Gil-González D. (2014). Social and immigration factors in intimate partner violence among Ecuadorians, Moroccans and Romanians living in Spain. *European Journal of Public Health*.

[47] Rahman M., Nakamura K., Seino K., Kizuki M. (2012). Intimate partner violence and use of reproductive health services among married women: evidence from a national Bangladeshi sample. *BMC Public Health*.

[48] Rahman M., Hoque M. A., Makinoda S. (2011). Intimate partner violence against women: is women empowerment a reducing factor? a study from a national bangladeshi sample. *Journal of Family Violence*.

[49] Murshid N. S., Critelli F. M. (2017). Empowerment and intimate partner violence in pakistan: results from a nationally representative survey. *Journal of Interpersonal Violence*.

[50] McCloskey L. A., Williams C., Larsen U. (2005). Gender inequality and intimate partner violence among women in Moshi, Tanzania. *International Family Planning Perspectives*.

[51] Boyle M. H., Georgiades K., Cullen J., Racine Y. (2009). Community influences on intimate partner violence in India: Women's education, attitudes towards mistreatment and standards of living. *Social Science & Medicine*.

[52] Flake D. F. (2016). Individual, family, and community risk markers for domestic violence in Peru. *Violence Against Women*.

[53] Ahmed M., Chowdhury I. U., Laskar S. S. (2017). Domestic violence in relation to women empowerment and women household headship: a case in Nigeria. *Nile Journal of Business and Economics*.

[54] Kabir R., Majumder A. A., Arafat S. Y. (2018). Impact of Intimate Partner violence on ever married women and utilization of antenatal care services in Tanzania. *Journal of College of Medical Sciences—Nepal*.

[55] Akilova M., Marti Y. M. (2014). What is the effect of women’s financial empowerment on intimate partner violence in Jordan?. *Global Social Welfare*.

[56] Pambè M. W., Gnoumou B., Kaboré I. (2014). Relationship between women’s socioeconomic status and empowerment in Burkina Faso: A focus on participation in decision-making and experience of domestic violence. *African Population Studies*.

